# Acceptability of an online peer support group as a strategy to improve antiretroviral therapy adherence among young people in Kampala District, Uganda: qualitative findings

**DOI:** 10.21203/rs.3.rs-4269582/v1

**Published:** 2024-11-18

**Authors:** Yerusa Kiirya, Sabrina Kitaka, Joan Kalyango, Joseph Rujumba, Gloria Adobea Odei Obeng Amoaka, Mathew Amollo, Joan Nangendo, Charles Karamagi, Philipa Musooke, Anne Katahoire

**Affiliations:** Makerere University College of Health Sciences Clinical Epidemiology Unit; Department of Paediatrics, Makerere University College of Health Sciences,; Makerere University College of Health Sciences Clinical Epidemiology Unit; Department of Paediatrics, Makerere University College of Health Sciences,; Department of Nutrition and Food Science, School of Biological Sciences, College of Basic and Applied Sciences; Department of Social Work and Social Administration, Makerere University; Makerere University College of Health Sciences Clinical Epidemiology Unit; Makerere University College of Health Sciences Clinical Epidemiology Unit; Department of Paediatrics, Makerere University College of Health Sciences,; Child Health and Development Centre, School of Medicine, Makerere University College of Health Sciences

## Abstract

**Introduction:**

Peer support groups may contribute to adherence and play a role in decreasing stigma to antiretroviral therapy (ART) adherence among young people living with HIV (YPLHIV). However, peer support activities usually occur face-to-face in Uganda and elsewhere in Sub-Saharan Africa, and thus have structural limitations and may not be readily available when young people need them. Online peer support has the potential to help YPLHIV access regular psychosocial support without significant effort or cost. We assessed the acceptability of a WhatsApp-based peer support group as a strategy to improve ART adherence among Ugandan YPLHIV.

**Methods:**

We conducted a formative qualitative study in three health facilities in Kampala, Uganda, between July and August 2022. We held four focus group discussions with twenty-six YPLHIV seeking services at the study facilities. We also conducted six key informant interviews with health providers attached to adolescent HIV care clinics. The data was analyzed using thematic analysis guided by the acceptability framework to understand socio-cultural beliefs and perceptions towards utilizing WhatsApp-based peer support groups for HIV care.

**Results:**

Overall, the peer support group on WhatsApp was acceptable for use among YPLHIV. The young people regarded it as convenient because it would save time and would be more cost-effective compared to the transport costs of in-person meetings. Health providers revealed that the WhatsApp peer support group could reduce the stigma associated with community follow-up for non-adhering young people and empower YPLHIV to overcome stigma. Both the young people and health providers suggested that online peer support could provide accessible emotional support, which could improve YPLHIV’s psychosocial well-being and enhance adherence to ART. However, participants raised concerns about privacy, the cost of internet bundles, and smartphones, especially for younger adolescents.

**Conclusion:**

Online peer support groups are acceptable to Ugandan YPLHIV and hold promise in enhancing psychosocial support and improving treatment adherence in this sub-population. In implementing online support groups, due consideration should be given to software tools with high privacy standards and zero-rated data use for new apps. Research is needed to evaluate the feasibility and effectiveness of this peer support model in Uganda.

## Background

Adolescents and young people living with HIV (YPLHIV) account for 45% of new HIV infections globally, with 70% of this population residing in sub-Saharan Africa (SSA) [1]. Only 37% of YPLHIV in Sub-Saharan Africa on antiretroviral therapy (ART) have viral load suppression (VLS<1000), which is a priority for ending the HIV/AIDS epidemic by 2030. In Uganda, 54.7% of YPLHIV aged 15 to 24 years have VLS which can be ascribed to sub-optimal adherence to ART or virological resistance [2]. In Uganda, 67–87% of the YPLHIV adhere to treatment, and this is lower than in older age groups[3–5]. The sub-optimal ART adherence among YPLHIV results from complex personal, interpersonal, and contextual challenges [6]. Among these challenges are the psychosocial barriers exacerbated by the social cognitive development changes that occur during adolescence and young adulthood [7]. Social acceptance is more critical for this age group than for any other age group [4], yet many YPLHIV experience stigma and bullying, leading to negative self-images, low self-efficacy, anxiety, and depression [3, 6, 8–11]. Depressed YPLHIV are more likely to abuse alcohol and drugs[12].

The World Health Organization (WHO) and Ministry Of Health Uganda (MoH) recommend peer support groups to offer psychosocial support to YPLHIV[13, 14]. However, in Uganda and most SSA countries, peer support group activities occur face-to-face and most often in health facilities [15, 16]. This approach has limitations like the need to travel, inconvenient working hours, and inadequate safe space for psychosocial services [15, 17]. Face-to-face interactions were further limited when there were lockdowns during the COVID pandemics. Thus, psychosocial services may not be available when young people need them, and hence the need for more real-time and widely feasible interventions. With the rapid increase in mobile phone availability in SSA, online peer support groups have the potential to help YPLHIV access regular and timely support [18]. In Uganda, 60.7% of the young people own a mobile phone and 87.9% of these use them for social media [19, 20]. Social media platforms permit virtual communities and can serve as a place for peer support group activities[21].

Online peer support groups in the United States of America (USA) and China have improved psychosocial outcomes[22, 23], ART adherence[24–26], and VLS among people living with HIV/AIDS (PLHIVA) [25, 27, 28]. However, studies in Sub-Saharan have provided mixed results with no effect on psychosocial outcomes [29–31], but also increased stigma levels[32]and no significant effect on ART adherence among YPLHIV[29, 31]. Acceptability is a necessary condition for effectiveness of a health care intervention [33]. Successful design, implementation, and evaluation of a health care intervention depends on the acceptability of the intervention to both deliverers and recipients. Although a few acceptability studies in SSA have shown promising results [31, 34, 35], they were not guided by theory or theoretical frameworks. Furthermore, precisely what people find acceptable is deeply contextualized and interlinked with prevailing social and cultural norms[36]. Understanding and designing for such norms is critical to the success of online peer support groups yet these remain unknown for YPLHIV in Uganda. Sekhon etal 2017, defined acceptability as a multi-faceted construct that reflects the extent to which people delivering or receiving a healthcare intervention consider it to be appropriate, based on anticipated or experienced cognitive and emotional responses to the intervention[33]. Acceptability can be assessed at three levels: prospectively before intervention enrollment, concurrently while the intervention is being implemented and retrospectively following implementation of an intervention[33]. In this study, we applied the Theoretical Framework of Acceptability of health care interventions by Selkon etal, [33] to prospectively explore the acceptability of using a WhatsApp peer support group as a strategy to improve ART adherence among YPLHIV in Uganda.

### Description of intervention

In addition to receiving the standard of care, YPLHIV will be enrolled in a WhatsApp group with peer counsellors. We will allocate YPLHIV to a WhatsApp group depending on their age (15–18 years vs 19–24 years) and the health facility where they seek care. In the WhatsApp group YPLHIV will communicate individually and as a group with their peer counsellors through private messages and the group chat. The study team will share weekly educational videos on different topics based on the Adolescent Treatment Literacy Guide for Support Group Settings[37] and the Ministry of Health’s HIV care guidelines[14]. The study team will use social listening to identify gaps in messaging and care concerns on the group chat. These will inform the videos and discussions in the next week. The other aspect of the group is private communication as direct chats with peer counselors at least once a week. The messages will be tailored to young people’s treatment schedules, clinic appointments, and psychosocial state. However, young people can text a peer counselor whenever they wished to do so. The standard of care includes health education sessions, screening and treatment for infections, and psychosocial services. Psychosocial services offer peer support, counselling, home visits, and referrals based on individual needs. YPLHIV are evaluated for their adherence to ART and VLS. Non-suppressing young people undergo intensified adherence counselling (IAC).

## Methods

### Study site and population

We conducted a formative qualitative study to explore the perspectives of study participants on the potential use of a WhatsApp peer support group as a strategy to improve ART adherence among YPLHIV. The study was conducted at three health facilities in Kampala capital city. Kampala is divided into five divisions, with over 391,065 young people living in this city[38]. The literacy level among young people in Kampala is approximately 61% and only 41.4% are employed [39]. More than half of the young people in Kampala city access the internet, 76% own cell phones, and 87.9% use their cell phone for social media [20]. In Kampala, 3.4% of the young people are living with HIV, and 72% of these access treatment [40]. Kampala has 93 HIV care facilities, of which 23 (25%) are owned by the government, with the majority (13/23) under Kampala City Council Authority (KCCA) management[41].We purposively selected a KCCA health facility with the highest number of YPHIVA in three divisions namely Kawaala HCIV in Lubaga Division, Komamboga HCIII in Kawempe Division, and Kiswa HCIII in Nakawa Division.

The facilities serve a semi-urban population and over 1,800 young people (15–24 years) had received ART by March 2020 [41].

### Data collection

The data was acquired through conducting Focus Group Discussions (FGDs) with YPLHIV and Key Informant Interviews (KIIs) with health providers.

### Key Informant Interviews

We purposively selected health providers with expertise in providing HIV care to adolescents and young adults to participate in the key informant interviews (KIIs). We included health providers from study sites and affiliated organizations with more than one year’s working experience with YPLHIV. Health providers were approached in person during clinic days to participate in the study. Six health providers took part in the KIIs, and these included two clinical officers, one nursing officer, one community liaison officer, and two counselors attached to YPLHIV clinics at the study sites. KIIs were held privately in counselors’ and clinical officers’ rooms. The principal investigator (PI) conducted all the KIIs in English with a semi structured topic guide. The topic guide was developed by the PI and reviewed by all members of our multi-disciplinary research team including an experienced adolescent health and young adults specialist (SBK), a paediatric infectious disease specialist with particular interest in HIV research (PM), two epidemiologists and biostatisticians (JK and CK), and a social behavioral scientist with expertise in qualitative research (ARK). The development of the topic guide was informed by current knowledge about online peer support groups, health workers’ experiences with implementing interventions for YPLHIV and our research questions. The topic guide was pilot tested on an adolescent program coordinator and a medical doctor at Mulago Immune Suppression Syndrome (**ISS**) Clinic Kampala. The interviews lasted between 35 to 50 minutes and were all audio recorded.

### Focus group discussions

We purposively selected twenty-six YPLHIV aged 15–24 years from clients registered in the ART clinics at the study sites to participate in focus group discussions. First, we reviewed YPLHIV records to screen for study eligibility and obtain their contact information. We included YPLHIV who knew their HIV status and were enrolled in HIV care 12 months before study entry. We excluded those in boarding school and those enrolled in other studies. Eligible YPLHIV were invited to take part in the Focus Group Discussions (FGDS) either during their clinical visits or by phone. The purposive selection also ascertained representative and collective input from participants based on their age, sex and marital status. We held four focus group discussions each with 6–9 participants. We grouped study participants based on their age (two FGDs with adolescents aged 15–19 years and two FGDs with young adults aged 20–24 years) and facility (two FGDs from each facility Kawala HCIV and Komamboga HCIII). The PI conducted the FGDs privately in each ART clinic in Luganda, the most widely spoken local language in the area, using a translated discussion guide. The development of the discussion guide was similar to that described for the KII topic guide above except that it was informed by YPLHIV attitudes towards online peer support and pilot tested among five YPLHIV at Mulago ISS. Guides were flexible and modified as needed during the study. The discussions lasted between 58 to 80 minutes. At the end of every topic of discussion the PI summarized and confirmed her interpretation of what members said. All discussions were audio recorded and field notes were taken upon completion of the discussions.

Data collection for both KIIs and FGDs was an interactive process. The PI reviewed recordings of the initial group discussions and interviews to identify gaps that needed to be filled in future discussions and interviews. Data collection stopped after four FGDs and five KIIs because no new information emerged from the interactions.

### Theoretical framework guiding analysis

We used the theoretical framework of acceptability (TFA) of health care interventions[33] to guide our analysis. TFA consists of seven constructs, namely affective attitude, burden, perceived effectiveness, ethicality, intervention coherence, opportunity costs, and self-efficacy[33]. Affective attitude refers to how an individual feels about the intervention. Burden includes the amount of effort required to participate in the intervention. Opportunity cost is the extent to which benefits, profits, or values one must give up to engage in the intervention. Perceived effectiveness is the extent to which the intervention is perceived to achieve its purpose. Ethicality is the extent to which the intervention aligns with the value system of the participating individual. Intervention coherence refers to the extent to which the participant understands the intervention. Finally, Self-efficacy is the participant’s self-confidence that they can perform the behaviors that are required for intervention participation[33]. For this study we focused on the anticipated cognitive and emotional responses to the intervention.

### Data management and analysis

Data were transcribed verbatim by a research assistant and Luganda transcripts were translated into English. The PI proofread all transcripts, comparing them to the audio recordings before sharing them with the data analyst, a health services researcher with over 15 years’ experience of qualitative research. The data analyst and PI reviewed the transcripts for completeness, formatted them, and removed any identifying details. A unique identifier was assigned to each transcript to conceal the information sources and any text within the body of the transcripts with identifying information like names was removed before coding and analysis. This process was followed by the development of a codebook which was developed after reading through the first two FGDs and first three KII transcripts. The PI and the data analyst independently reviewed the first three transcripts and each developed separate codes. The PI and the data analyst met to harmonize the codebook, code definition, and structure. The harmonized codebook was then deployed for use during the data coding of this research work. This codebook comprised of preliminary sub-thematic areas, which were refined as other transcripts were coded and new themes identified. The PI and data analyst reviewed the coding structure for each theme and refined the codebook by comparing and categorizing emerging themes and their definitions. We followed the seven constructs of the Theoretical Framework of Acceptability of healthcare interventions to categorize the subthemes. TFA was chosen because it defines acceptability clearly and has been used widely in health science research. Towards the end of the analysis of the qualitative data, a member of the wider research team (ARK) examined the transcripts which had been coded by the PI and data analyst most closely involved in data collection and analysis, as an independent check on the assignment of codes to data. Coding and analysis of these data were conducted with the help of Atlas ti version 2023.

## Results

### Participants’ characteristics

Twenty-six YPLHIV currently seeking care at the study site participated in the study. Most of them were above 19 years of age (54%), over half (58%) of the participants were female and 54% were single (Table 1). Six health care professionals participated in the study, with a male to female ratio of 1:5. Four health care professionals (HCPs) had been working with adolescents for five or more years and the majority had a Bachelor’s degree as their highest level of education (n = 4) Table 2.

The study findings are reported following the consolidated criteria for reporting qualitative research (COREQ), under constructs of the TFA as shown in Table 2.

### Affective attitude

Overall, most participants found the WhatsApp peer support group convenient and were eager to join, resulting in positive feedback. They viewed convenience as saving time and transport costs. Young people believed that WhatsApp peer support groups were more cost-effective than in-person ones due to transportation expenses.

“It helps when I do not have transport; I get one thousand shillings for data and go to WhatsApp. I can chat through my problem rather than getting too much money for transport”

(Female 21 years FGD2 Kawala HCIV)

Furthermore, unlike in-person meetings, WhatsApp peer support allows information to be discussed without being physically present.

“Someone may not get transport, so cannot attend the in-person meetings. He/she misses the peer-to-peer educative session! With WhatsApp, I can still access the information at any time”

(Female 20 yrs FGD2 Kawala HCIV)

Young people appreciated WhatsApp peer support groups for quick responses, saving them time typically spent travelling and waiting at the facility.

“They ask us to come early at nine o’clock when they have organized a meeting. But on WhatsApp, I can post whatever question or concern I have in the morning, go to school, …come back later in the evening and find the advice I need. It will save us a lot of time”

(Female 20 years FGD2 Kawala HCIV).

Clinicians agreed that using a WhatsApp group is a convenient way to offer support to young people.

“It does not need someone to walk from home to the facility it saves transport. It saves time and someone is free to express himself or herself. So, it will help these young people a lot….”

(KI Clinical Officer Kiswa HCIII).

Clinicians and counselors perceived WhatsApp peer support groups as a potentially effective communication platform for and among young people. They gave various reasons why it could potentially be effective, such as quick responses, availability when young people need to communicate, personalized and group communication for specific concerns.

“….. the assurance that I have someone I can contact in case I am having an issue. I can inbox my issue privately if I don’t want to share it with the group and I can get a quick response”

(KI Community Liaison Officer Komamboga HCIII).

### Perceived effectiveness

The study participants were optimistic about the effectiveness of WhatsApp peer support groups in providing social support and improving adherence to antiretroviral therapy (ART). However, each group reported unique benefits from this mode of peer support. Young people considered the WhatsApp peer support groups as a readily available source of social support, particularly for receiving both informational and emotional support.

“When we have our WhatsApp group as young people in Komamboga. We will find time to talk. We will teach each other some things”

(Female 15 years FGD1 Komamboga HCIII).

Young people perceived the WhatsApp peer support group as a safe space where they could share challenges and find support.

“We have hurting experiences, but we cannot share them with people who do not know our HIV status. But if you have someone who knows your situation, you can share it with them and they find a solution”

(Female 24 years FGD1 Komamboga HCIII).

Young people reported that this support might reduce feelings of isolation and worry, thus improving their psychosocial well-being.

“it will help us encourage ourselves and not to worry a lot…………. To stop worrying, some commit suicide when they feel alone. With the WhatsApp group, young people will be encouraged “knowing there are people who care. So they may not have suicidal ideations”

(Female 21 years” FGD2 Kawala HCIV)

In addition, young people acknowledged that virtual support groups were a suitable form of emotional support when in-person meetings are restricted.

“WhatsApp group is a good idea; you may have a challenge but have no one to talk to at home. Yet with the WhatsApp group, you can share with members and get advice”

(Female 24 years FGD1 Komamboga HCIII).

Young people reported that the readily available emotional support on the WhatsApp peer support group could improve ART adherence.

“We will encourage ourselves to take drugs and good care of ourselves”

(Female 24 years FGD1Kawala HCIV).

Counselors and clinicians had similar opinions, counsellors suggested that virtual support groups could motivate young people to adhere to treatment.

“I think the WhatsApp group can motivate YPLHIV to take their drugs, it can help us achieve our goal of encouraging them to adhere to treatment”

(KI Counsellor Komamboga HCIII).

In addition, clinicians considered the WhatsApp peer support group a better intervention for improving ART adherence among young people because it encourages freedom of expression.

“I believe it’s a better intervention in improving adherence among young people, since they are free to express their feelings on WhatsApp. So I believe it’s going to work”

(KI Clinical Officer Kiswa HCIII).

Counselors saw the freedom of expression as an important condition in empowering young people with the knowledge needed for ART adherence.

“I think it will empower them with knowledge because they will discuss several topics. And if they have the knowledge, it will improve adherence”

(KI counselor Kawala HCIV).

Relatedly counselors reckoned through WhatsApp peer support groups young people will access less stigmatizing adherence reminders and thus might improve ART adherence.

“…young people will open their WhatsApp and the messages will remind them to take their medicine. And there is that confidentiality where someone is not going to say, eh!….. please, when are taking your medicine”

(KI counselor Komamboga HCIII).

Counsellors further acknowledged that WhatsApp peer support groups could empower young people to overcome stigma.

“It will empower them to fight against stigma, they will learn from one another as they are chatting”

(KI counselor Kawala HCIV).

In addition, virtual support could reduce the stigma associated with community visits; the current approach for following up young people with adherence challenges in Uganda.

“It reduces the level of stigma because when you send teams to look for young people who miss their clinical appointment or are non-suppressing. The teams have to introduce themselves… I am so and so.….I am looking for Jane because she is our client. When you send a WhatsApp message, no one will know and the young person will not be stigmatized.”

(KI counselor Komamboga HCIII).

However, one counselor was of the view that WhatsApp peer support groups may not be effective in providing psychosocial support and improving ART adherence, given that not all young people can afford the data and smart phones required to take part in the WhatsApp peer support group.

“If a counselor is online, what are the chances that he/she will find everyone on the platform at the time of discussion? You might find that someone is off the platform because of data issues, because either the phone is not theirs”

(KI Community Liaison Officer Komamboga HCIII)

### Burden;

Under this TFA construct, we present the participants’ perspectives on the burdens that the intervention would place on YPLHIV. We focused on the time, expenses, and cognitive effort required to take part in the WhatsApp peer support group. Young people emphasized the high cost of data needed for the WhatsApp peer support groups. They complained about the chances of +failing to share a problem because one does not have data.

“someone might have a problem when they don’t have money for data”

(Male 19 years FGD1 Komamboga HCIII).

Clinicians and counselors had similar concerns that some young people may not consistently access the WhatsApp peer support groups because of the high cost of data.

“The challenge is that some young people may not be online because they have to depend on their parents for data”

(KI Clinical Officer Kiswa HCIII).

Young people further highlighted the fact that smartphones needed for WhatsApp peer support groups are expensive.

“The biggest challenge is that some of us don’t own smartphones”

(Female 15 years FGD2 Komamboga HCIII).

To overcome this challenge, some young people suggested sharing phones with their parents. However, the process of accessing parents’ smartphones might be lengthy and frustrating.

“If you don’t have a phone, you can use your parents’ phone, if they know your status. If you have not disclosed, you look for ways of getting one”

(Female 20 years FGD2 Kawala HCIV)

“Remember, sometimes you may fear asking for it when he/she is using it for business, and then you claim you want to talk to the group”

(Female 18 years FGD Kawala HCIV).

So, adolescents were less likely to participate in the WhatsApp peer support group discussions since they have to seek permission from their parents/guardians and might access the phones for limited hours.

“For an adolescent who doesn’t own a smart phone it will be very difficult for them to be active”

(Female 24 years FGD1 Kawala HCIV)

Counselors and clinicians held the same view that young adults could afford smartphones needed for WhatsApp but not adolescents.

“the age bracket of 20–24 most of them have smart phones. The only limitation is that adolescents may not afford smart phones”

(KI Clinical Officer Kiswa HCIII)_

Nonetheless, counselors’ perceived WhatsApp peer support groups as a more cost-effective approach of following up with YPLHIV compared to traditional face-to-face approaches.

“With WhatsApp, you will send a message and the young person will read it. I think it’s easier than sending someone because community follow-ups are expensive. I think they cost 120,000 Ushs per week and even the risks associated with community follow-ups are very high”

(KI counselor Komamboga HCIII).

### Opportunity costs

Under this construct participants revealed the extent to which benefits, profits, or values must be given up for young people to engage in the WhatsApp peer support groups. A common thread among young people, counselors, and clinician interviews was the concern about the risk of breach of confidentiality associated with WhatsApp peer support groups. Participants expressed concerns about the possibility of third parties accessing client information through phone sharing, or through unrestricted access to the WhatsApp group information. This risk was perceived to be greater among young people who share phones with siblings or with other adults. This is what participants had to say:-

“This is what I was thinking about breach of confidentiality where may be certain discussions are accessed by non-group members”

(KI counselor Kawala HCI V)

“Some of us share our phones, now someone may request for your phone and goes to your chats…..!”

(Male 23 years FGD1 Komamboga HCIV)

So young people recommended personal identification numbers of any restrictive patterns or thumbprints to limit unauthorized phone and WhatsApp users.

“We can protect our phones by limiting access, and adding a password to the phone and group itself that no one knows”

(Female 15 years FGD1 Komamboga HCIII)

Health providers identified cyberbullying as an issue with the WhatsApp peer support groups that needed to be dealt with.

“Then this issue of bullying;…. Someone might say something that affects another young person in a negative way”

(KI Community Liaison Officer Komaboga HCIII).

However, young people did not perceive potential bullying as a challenge of the WhatsApp peer support group.

“We need the WhatsApp peer support group. We cannot abandon it because of bullies. We have bullies at school but you do not quit because of a bully”

(Male 15 years FGD1 Kawala HCIV).

### Ethicality of the intervention

In this construct, we focused on the extent to which the WhatsApp peer support group was perceived to be a good fit for YPLHIV value systems. Ethical concerns raised by participants were classified into equity, literacy skills, and morality concerns. Regarding morality, all health providers were concerned that WhatsApp peer support groups could escalate romantic relationships among young people. This is what a counsellor had to say;-

“Young people want to explore. They can catch up and engage in relationships”

(KI Counselor Kawala HCIV)

A similar observation was highlighted by some young people who had mixed sentiments to unsolicited romantic relationships. They recommended development and implementation of standards of behavior for group members, which included no tolerance to coupling among group members.

“No “coupling”, those in love, should express themselves privately, but not in the group. Those coupling should be dismissed from the group”

(Female 24 years FGD1 Komamboga HCIII).

A few young people indicated parents would be reserved to give mobile phones to their children to take part in the WhatsApp peer support group for fear that their children will access pornographic content.

“…. when you talk about the phone, as if you have told her/him to chase you from home…. He/she thinks you are going to pornography now”

(Male 16 years FGD1 Kawala HCIV)

All participants were concerned that WhatsApp peer support groups would exclude young people who could not afford or access a smartphone and those in boarding school. Below are some of the voice excerpts from the participants:

“It is good but then what about those who don’t have smart phones? Won’t they miss out?”

(Female 21 years FGD2 Kawala HCIV).

…“You did not think about adolescents in boarding schools”

(KI Clinical Officer Kiswa HCIII).

One clinician indicated that young people who cannot afford smartphones often have more severe psychosocial needs. This clinician had this to say:

“You will exclude children from my experience who are depressed, non-suppressing, and abused. They are the ones who cannot access social media”

(KI Clinical Officer Kiswa HCIII)

Counselors mentioned WhatsApp peer support groups require literacy skills, which might be limited among young people.

“It requires you to know how to read for you to understand the discussions. You can use Luganda for easy interpretation but still, some young people may not understand the information…”

(KI Community Liaison Officer Komamboga HCIII)

During discussions, young individuals agreed that some of their peers lacked typing skills and suggested using audio messages to encourage their participation in the intervention.

“…. if you don’t know how to type, you don’t know English, you may use Luganda. If you can’t use Luganda like me, I think you may use audio messages”

(Female 20 years FGD2 Kawala HCIV).

Nonetheless, the intervention was considered suitable for the lifestyles of young individuals. All healthcare providers agreed that WhatsApp peer support groups were an ideal match for young people who have embraced social media. These participants noted that young people were more likely to benefit from the WhatsApp peer support group due to the high adoption of social media and the benefits provided by social media platforms, such as the freedom of expression and the ability to disclose issues they may not discuss during in-person meetings. Here is what some of the participants had to say:

“It’s good it will help those who can access social media because young people love WhatsApp. When they are discussing with fellow young people, they open up more than with health workers or parents”

(KI Clinical Officer Kiswa HCIII).

“I will get to know the individual issues young people have.. challenges …..they face but are not free to share in face-to-face sessions”.

(KI Community Liaison officer Komamboga HCIII)

### Self-efficacy

Young people and consellors perceived organizing virtual meetings as easy and convenient compared to physical meetings. Young people indicated that the intervention empowers them to participate actively in organizing peer support sessions. One participant had this to say:

“It is difficult to get transport of 25000/= to reach here. However, with WhatsApp, we can suggest a day when we meet to discuss it makes life easier”

(Female 15 years FGD2 Komamboga HCIII).

### Intervention coherence

Young people shared their views regarding how they understood the virtual peer support group to work. To most young people, this intervention is not different from the various WhatsApp groups created to meet specific goals.

“I think you can easily get information on facility activities. They can post, for example, on Saturday we have a youth meeting. You just read the message and get the information”

(Male 19 years FGD2 Komamboga HCIII).

## Discussion

The research findings showed that WhatsApp peer support groups are considered beneficial and easily accessible by YPLHIV and health providers in Uganda. They valued how easy and convenient it is to communicate on this platform, which could offer immediate emotional support to YPLHIV, leading to improved psychosocial well-being and ART adherence. Health providers further revealed that the WhatsApp peer support group could reduce the stigma associated with community follow-up for non-adhering YPLHIV and empower them to overcome stigma. However, both young people and health providers were concerned about potential breaches of confidentiality, and the cost of smartphones and internet bundles, especially for the younger age group. Health providers further expressed concerns about the potential for online bullying and escalated romantic relationships through WhatsApp peer support groups. However, young people had mixed sentiments about unsolicited romantic relationships and did not consider cyberbullying a challenge for online peer support.

Consistent with findings reported by other studies in Sub-Saharan Africa online peer support groups are acceptable among YPLHIV in Uganda[32, 35, 36]. According to a Facebook study, YPLHIV in Nigeria prefer online peer groups over in-person ones [35]. Online peer support groups that use popular social media platforms like WhatsApp are particularly well-received and may have a better uptake compared to web-based interventions as shown in studies conducted in South Africa and Kenya[32, 36]. However, there were concerns about privacy and anonymity when sharing sensitive information with others in the group and the risk of a breach of confidentiality if a third party gains access to a member’s phone. Several studies also reported users’ concerns about the privacy and anonymity of online peer support groups that use social media platforms[46, 47]. Online peer support groups can offer privacy and anonymity through customized software development. Given the acceptance of online peer support groups and the growing access to social media, it is worth exploring their feasibility and effectiveness for YPLHIV in Uganda and other Sub-Saharan countries. If proven effective, the delivery of such groups at scale may necessitate developing and maintaining custom software with strong privacy and anonymity protections.

The acceptability of the WhatsApp peer support group can be attributed to several factors, with convenience being the most notable. These findings align with previous research which highlights the potential of online peer support groups to provide psychosocial support while overcoming barriers of time and location of service[46, 47].Thus could be used to provide immediate and accessible psychosocial services to YPLHIV. A review of technology-based health interventions for HIV-positive adolescents in low- and middle-income countries found several benefits of online peer support groups[47]. These include giving young people hope, improving morale, and creating a sense of community and support for adolescents living with HIV who may not have had access to these before[47]. They also reported several challenges, such as anonymity, confidentiality, encouraging the participation of all members, and literacy skills[47]. Some young people and health providers had similar concerns as reported by Crowley et al. 2023, including equity, morality, smartphone, and data costs[47]. Despite the cost of smartphones and data, our study found that online peer support groups are perceived as a cost-effective alternative to in-person meetings. Our findings reinforce the benefits of online peer support groups in optimizing psychosocial support among YPLHIV and highlight areas of caution during the implementation and future scaling up of these support groups.

Young people thought it would be easy to get social support through WhatsApp peer support groups. They appreciated the support they would receive, which would help them discuss their struggles and encourage one another in a safe space. This would reduce their loneliness and improve their emotional wellbeing. Studies conducted in Sub-Saharan Africa confirm online peer support groups can benefit YPLHIV in many ways, such as sharing knowledge and experiences, connecting with others, finding emotional support, and feeling recognized and understood [35, 36]. These have been linked to positive psychosocial outcomes [48] and online peer support groups for YPLHIV offer acceptance and a sense of normalcy [35, 36], which reduce isolation and improve emotional well-being [48]. Online peer support groups can offer several psychosocial benefits to YPLHIV with no structural limitations. Public health practitioners could consider adopting online peer support groups to optimize social support for YPLHIV in Sub-Saharan Africa.

The study revealed that WhatsApp peer support groups could reduce the stigma associated with community follow-up for non-adhering young people and may have the potential to empower YPLHIV to overcome stigma. Our results align with research in the US which showed that online peer support groups, empowered PLHIV to reject stigma and isolation through fostering positive self-images and supportive relationships[49]. Furthermore, Lesbian, gay, bisexual, transgender, and queer individuals with HIV found social support through online peer support groups when stigma and discrimination prevented them from seeking help in person[50]. On the contrary in Kenya Ashely et al. 2022, found that stigma levels increased among adolescents living with HIV as group discussions encouraged participants to acknowledge and share stigmatizing attitudes, beliefs, and experiences[33]. In Nigeria, no significant differences in HIV-related stigma were observed among YPLHIV enrolled in an online peer support group[30]. However, the studies conducted in Sub-Saharan Africa were small pilot studies with limited sample sizes[30, 33]. Online peer support groups may be’ an innovative way to reach and empower young people affected by HIV/AIDS and tackle stigma. The effect of online peer support groups on stigma among YPLHIV in Sub-Sahan Africa still remains unclear with mixed conclusions and multiple methodological caveats. Larger RCTs are necessary to reduce this knowledge deficit.

The study results suggest WhatsApp peer support groups could offer a multifaceted approach to ART adherence challenges among YPLHIV. WhatsApp groups could improve ART adherence among YPLHIV by offering emotional support, stigma-free reminders, and knowledge empowerment. Studies have shown that YPLHIV in different settings have similar preferences for mobile health interventions [22]. These include; those that provide credible, up-to-date information on HIV and general health and wellness, ART adherence reminders, and those that ease connections to providers and other YPLHIV[22]. Studies conducted in the USA and China found that. online peer support groups which used a multifaceted approach improved ART adherence[25, 27, 51]. However, evidence on online peer support groups for improving ART adherence in Sub-Saharan Africa is inconclusive due to small sample sizes and short follow-up periods[30, 32]. Therefore, it is yet to be determined whether online peer support groups can effectively improve ART adherence for this population.

### Strengths and limitations

This study has several strengths; first, using the theoretical frame work of acceptability facilitated a systematic inquiry which is vital at this stage of planning implementation. With the TFA, we could examine all aspects that might hinder the use of the WhatsApp peer support group. Our study explored the acceptability of a WhatsApp peer support group among YPLHIV and adolescent health providers who had not yet incorporated it into their regular practice. With this approach, the study generated feedback that can be used to address challenges ahead of time to improve the implementation and clinical outcomes of the intervention. Finally, we comprehensively understood the acceptability of a WhatsApp peer support group and validated study findings by using multiple data sources, such as FGDs and KIIs.

Notwithstanding these strengths, our study had limitations; Although we used the TFA, it was limited to analysis and presentation of study findings and not the design of the topic guides, thus we could have missed out on the systematic generation of knowledge about the intervention. In addition, the lead PI of the study designed the qualitative data collection tools and conducted all the KIIs and FGDs so it could have been very difficult for her to be totally objective and to set aside her personal interests, and thus taking an insider position[52]. However, several measures were taken to mitigate reflexivity bias including data triangulation, eliciting contributions from a broad range of adolescents, young people, and different cadres of health providers (Clinicians, nurses, counsellors and community workers) and team data analysis as described in the methods section above[52]. Finally, the study was conducted in three urban facilities thus the generalizability of findings may be limited to urban settings.

## Conclusion

The study findings suggest online peer support groups are generally acceptable to urban Ugandan YPLHIV. While YPLHIV in Uganda prefer online peer support groups that use popular social media platforms, these pose confidentiality and internet bundles cost challenges. So, in implementing these support groups, due consideration should be given to development of software tools with high privacy standards and zero-rated data use for new apps. Given that online peer support groups hold promise in improving treatment adherence and enhancing psychosocial support for YPLHIV, further research should be conducted to evaluate the feasibility and effectiveness of this peer support model as a means of enhancing ART adherence among YPLHIV in Uganda.

## Figures and Tables

**Table 1:** Descriptive characteristics of young people living with HIV in Kampala district, 2022

Variable	Number	Percentage
**Young people**		
**Age**		
15– 18	12	46%
19– 24	14	54%
**Sex**		
Male	11	42%
Female	15	58%
**Marital status**		
Single	14	54%
Married	12	46%
**Level of education**		
None	1	4%
Primary	9	35%
Secondary	15	58%
Tertiary	1	4%
**Occupation**		
cleaner	1	4%
Business person	5	19%
Hair dresser	5	19%
House help	1	4%
Waitress	2	8%
Student	9	35%
None	3	12%
**Health care Profesinoal**		
**Sex**		
Male	1	17%
Female	5	83%
**Occupation**		
Clinical officer	2	33%
Nursing officer	1	17%
Counsellors	2	33%
Community Liassion officer	1	17%
**Highest level of education**		
Diploma	2	33%
Bachelors degree	4	67%

**Table 2:** Adapted TFA constructs and codes identified from the analysis.

TFA construct	Emerging themes
**Affective attitude**	Addresses transport challenges
	Time-saving
	An effective platform for timely information sharing among young people
	Suitable where there are restrictions/limitations to in-person meetings
**Perceived effectiveness**	Emotional support
	Improves ART adherence
	Potentially minimizes stigmatization
**Burden**	Data as a challenge
	Smartphone access
	Cost-effective
**Opportunity cost**	Confidentiality and privacy concerns
	Bullying
**Ethicality**	Morality
	Equity
	Literacy skills
	Good fit for youth lifestyles
**Self efficacy**	Easy and convenient
**Intervention coherence**	Similar to WhatsApp groups created for specific purposes
	Effective in mobilizing and sharing information among young people

## Data Availability

Data generated and analysed during this study are not publicly available due to potential breach of confidentiality, but are available through School of Medicine higher degrees research ethics committee at Makerere University College of Health Sciences via email (rresearch9@gmail.com) on reasonable request. The data obtained is in the form of audio recordings and verbatim transcripts, which are very difficult to remove all personal identifiers.

## Abbreviations

AIDS: Acquired Immunodeficiency Syndrome
ART: Antiretroviral therapy
HIV: Human Immunodeficiency virus
IDI: In-depth interview
MoH: Ministry of Health Uganda
PLHIVA: People Living with HIV/AIDS
TFA: Theoretical Framework of Acceptability
UNAIDS: The Joint United Nations Programme on HIV/AIDS
VLS: Viral Load Suppression
WHO: World Health Organisation
YPLHIVA: Young People Living With HIV
